# A feedback loop of PPP and PI3K/AKT signal pathway drives regorafenib-resistance in HCC

**DOI:** 10.1186/s40170-023-00311-5

**Published:** 2023-12-18

**Authors:** Huihua Yang, Dahong Chen, Yafei Wu, Heming Zhou, Wenjing Diao, Gaolin Liu, Qin Li

**Affiliations:** 1 Department of Clinical Pharmacy, Shanghai General Hospital, Shanghai Jiao Tong University School of Medicine, Shanghai, China; 2 Department of Pharmacy, Zhongshan Hospital, Fudan University, Shanghai, China

**Keywords:** Pentose phosphate pathway, Glucose-6-phosphate dehydrogenase, PI3K/AKT signaling pathway, NAD kinase, Regorafenib, Hepatocellular carcinoma, Metabonomics

## Abstract

**Background:**

Hepatocellular carcinoma (HCC) is a principal type of liver cancer with high incidence and mortality rates. Regorafenib is a novel oral multikinase inhibitor for second-line therapy for advanced HCC. However, resistance to regorafenib is gradually becoming a dilemma for HCC and the mechanism remains unclear. In this study, we aimed to reveal the metabolic profiles of regorafenib-resistant cells and the key role and mechanism of the most relevant metabolic pathway in regorafenib resistance.

**Methods:**

Metabolomics was performed to detect the metabolic alteration between drug-sensitive and regorafenib-resistant cells. Colony formation assay, CCK-8 assay and flow cytometry were applied to observe cell colony formation, cell proliferation and apoptosis, respectively. The protein and mRNA levels were detected by western blot and RT-qPCR. Cell lines of Glucose-6-phosphate dehydrogenase(G6PD) knockdown in regorafenib-resistant cells or G6PD overexpression in HCC cell lines were stably established by lentivirus infection technique. G6PD activity, NADPH level, NADPH/NADP^+^ ratio, the ratio of ROS positive cells, GSH level, and GSH/GSSG ratio were detected to evaluate the anti-oxidative stress ability of cells. Phosphorylation levels of NADK were evaluated by immunoprecipitation.

**Results:**

Metabonomics analysis revealed that pentose phosphate pathway (PPP) was the most relevant metabolic pathway in regorafenib resistance in HCC. Compared with drug-sensitive cells, G6PD enzyme activity, NADPH level and NADPH/NADP^+^ ratio were increased in regorafenib-resistant cells, but the ratio of ROS positive cells and the apoptosis rate under the conditions of oxidative stress were decreased. Furthermore, G6PD suppression using shRNA or an inhibitor, sensitized regorafenib-resistant cells to regorafenib. In contrast, G6PD overexpression blunted the effects of regorafenib to drug-sensitive cells. Mechanistically, G6PD, the rate-limiting enzyme of PPP, regulated the PI3K/AKT activation. Furthermore, PI3K/AKT inhibition decreased G6PD protein expression, G6PD enzymatic activity and the capacity of PPP to anti-oxidative stress possibly by inhibited the expression and phosphorylation of NADK.

**Conclusion:**

Taken together, a feedback loop of PPP and PI3K/AKT signal pathway drives regorafenib-resistance in HCC and targeting the feedback loop could be a promising approach to overcome drug resistance.

**Supplementary Information:**

The online version contains supplementary material available at 10.1186/s40170-023-00311-5.

## Background

Hepatocellular carcinoma (HCC), a principal type of liver cancer with high incidence and mortality rates, is mainly induced by hepatitis B and C virus, alcohol, metabolic Syndrome, Aflatoxin B1 and tobacco [1]. At present, surgical interventions and systemic therapies are the dominating management for HCC. Eliminating resection and liver transplantation, patients with advanced HCC could only anchor their hope on systemic therapies [2].

At present, sorafenib and lenvatinib for first-line therapy, regorafenib and nivolumab for second-line therapy are FDA approved drugs for advanced HCC[3]. Regorafenib is a novel oral multikinase inhibitor potently targeting vascular endothelial growth factor receptor, tyrosine kinases, platelet-derived growth factor receptor and fibroblast growth factor receptor etc. [4]. Regorafenib was approved for patients with advanced HCC who resistant to sorafenib in 2017, becoming a breakthrough of second-line therapy for HCC, which could improve the median survival time of sorafenib-resistant patient [5, 6]. However, drug resistance of regorafenib has been reported in the past few years. BCL-xL/MCL-1 [7, 8], Pin1 and Gli1/Snail/E-cadherin signaling pathway [9], sphingosine kinase 2 [10], and Wnt and TGF-β Signaling [11] have been reported to be associated with regorafenib resistance. Therefore, the prognosis of HCC is far from optimistic and it’s urgent to illuminate the mechanism of regorafenib resistance.

Currently, metabolic reprogramming is considered to be one of the hallmarks of tumorigenesis and supports the growth of cancer cells, resulting in resistance to chemotherapeutic drugs. In general, the metabolic profiling of drug-resistant cells are further modified compared to drug-sensitive cells, and metabolic changes including increased glutamine demand, aerobic glycolysis, capacity of redox, and the pentose phosphate pathway (PPP), activation of fatty acid oxidation, etc. [12]. It is reported that hexokinase 1 promoted resistance to regorafenib in HCC cells [13]. During the establishment of regorafenib-resistant cell lines, different colors of culture medium and mounted demand for nutrition were observed in regorafenib-resistant cells. Therefore, we hypothesized that the metabolism of regorafenib-resistant cells has been changed. However, investigation about the metabolic profiling of regorafenib-resistant cells remains clean slate. Metabolomics is a powerful tool for comprehensive research on characterization of metabolites in biological systems and is widely applied in drug discovery and precision medicine [14]. Untargeted metabolomics is a commonly used method to comprehensively evaluate metabolites and reveal unknown metabolic changes [15]. In this study, we performed untargeted metabolomics to illustrate the metabolic profiling of regorafenib-resistant cells in HCC and explored the mechanism of regorafenib-resistance based on metabolic reprogramming.

## Methods

### Chemicals and materials

The key chemicals and materials were listed in the Table S1.

### Cell culture

Huh7 and Hep3B cell lines were purchased from Institute of Biochemistry and Cell Biology, Chinese Academy of Sciences. All cells were maintained in Dulbecco’s modified Eagle’s medium (DMEM) supplemented with 10% fetal bovine serum(FBS) and 1% penicillin/streptomycin in a humidified atmosphere with 5% CO_2_ at 37 ℃.

### Establishment of stable regorafenib-resistant cell lines

The combination of intermittent and gradual increased dose therapy of regorafenib was used to establish regorafenib-resistant cell lines in HCC, namely Huh7-RR and Hep3B-RR. Huh7-RR and Hep3B-RR cells were trained up to 6 μM and 2 μM regorafenib, respectively. Cells were cultured in drug-free media at least for 24 h before experiments to prevent acute drug effects.

### Colony formation assay

1 × 10^3^ or 5 × 10^3^ cells per well were seeded in 6-well plates for 24 h and then treated with or without specific compounds for 48 h. After cultured in drug free medium for another 5 days, cells were stained with 0.1% crystal violet staining solution.

### Cell counting kit-8(CCK-8) assay

Cell viability was measured by cell counting kit-8 assay (CCK-8). Cells were seeded in 96-well plates at a density of 5 × 10^3^ cells per well. Cells were cultured in drug-free medium for 24 h before they were treated with gradient concentration of specific drugs. Then, 110 μl cell culture medium with 10% CCK-8 assay solution were added to each well and incubated for 1 h at 37 °C in cell incubator and measure absorbance (450 nm) using a microplate reader. Then inhibition rates were used to calculate half maximal inhibitory concentration(IC_50_) by GraphPad Prism 8.0 with the method dose-response-inhibition/log (inhibitor) vs. response-variable slope (four parameters).

### Flow cytometry (FCM)

Annexin V FITC Apop Dtec Kit I (BD Pharmingen, USA) and Annexin V-APC/7-AAD apoptosis kit(MultiSciences, China) were used to detected the cell apoptosis. Cells were seeded in 6-well plates at a density of 2 × 10^5^ cells per well for 24 h. Then cells were treated with regorafenib for 24 h and harvested in a microcentrifuge tube. After washed with cold PBS three times, cells were suspended in 100 μl binding buffer. 5 μl FITC (or APC) and 5 μl PI ( or 10 μl 7-AAD) were added to each tube and maintained for 15 min protecting from light before another 400 μl binding buffer were added. Finally, cell apoptosis were tested by BD_Accuri_C6 flow cytometry.

### Sample preparation for LC-MS/MS analysis

Huh7 and Huh7-RR cells were seeded in 8 different culture plates, respectively. 1 × 10^6^ cells/sample were harvested and snap freezed in liquid nitrogen upon extraction and stored immediately at – 80 °C. Thaw the cell samples slowly in ice and add 500 μL methanol before vortexing for 5 min. Snap freeze samples in liquid nitrogen for 3 min and vortex samples for 2 min after thawed in ice for 10 min and repeat the above operation twice. Centrifuge sample for 10 min at 4 ℃ at 12000 rpm to remove any insoluble material. Collect 200 μL supernatant and add 300 μL methanol with internal standard substance. Vortex samples for 1 min and keep them in – 20 ℃ for 1 h. Centrifuge sample for 15 min at 4 ℃ at 12000 rpm and collect supernatant for LC-MS/MS analysis.

### Chromatography

UPLC analysis performed on an Agilent 1290 Infinity LC HPLC system (Agilent Technologies, Wilmington, USA). Chromatographic separation was acquired on Waters T3 C18 column (100 × 2.1 mm, 1.8 μm; C18 MWM-13). The detection conditions were as follows: wavelength, 347 nm; flow rate, 0.35 mL/min; column temperature, 40 ℃; sample volume, 2 μL. The elution solution was water comprised 0.04% formic acid and acetonitrile. And the gradient solution procedure for acetonitrile was: 0.0~10.0 min: 95~5%; 10.0~11.0 min: 5~5%; 11.1~14.0 min: 95~95%.

### Mass spectrometry

Mass spectrometry was achieved on a QTOF/MS-6545 mass spectrometer (Aglient, CA, USA) with an electrospray ionization (ESI) interface. The scan range was from m/z 100 to 1000. Nitrogen (N_2_) and helium (He) were respectively used as the sheath and auxiliary gas and collision gas and. The acquisition parameters were as follows: ion spray voltage floating,+2500/− 1500 V; nebulizer gas pressure, 40 V; Fragmetor potential, 135 V; gas temperature, 325 ℃; sheath temperature, 325 ℃; gas flow, 8.0 L/min; Analyst QS 2.0 (Applied Biosystems/MDS Sciex) was applied for data acquisition and processing.

### Metabolomics analysis

LC-MS/MS raw data was transferred into mzXML format utilizing ProteoWizard. Then peak extraction, alignment and retention time align were processed through the online platform XCMS. Support vector regression was used to calibrate peak area. Peaks with miss rate greater than 50% were filtered out. Principal component analysis (PCA), partial least squares-discriminant analysis (OPLS-DA), Student’s *t* test, and fold change analysis were achieved in R program. The selected peaks was identified based on m/z value, mass spectra fragments, and retention behavior through metDNA (http://metdna.zhulab.cn/), HMDB (https://hmdb.ca/), and PubChem (https://pubchem.ncbi.nlm.nih.gov/). In addition, Kyoto Encyclopedia Genes and Genomes (KEGG, http://www.genome.jp/kegg/) analysis were performed on R program.

A qualitative control (QC) sample, created from equal-volume supernatant of all samples, was applied to detect the stability and repeatability of the instrument. In this study, a QC sample was injected every 8 samples throughout the entire procedure. Good superposability of total ion chromatogram (TIC) of QC samples was acquired with good consistent states of the retention time and the ion intensity in both positive or negative model (Figure S1A). Besides, different injections QC sample were clustered together(Figure S1B, blue spots). Therefore, stability and repeatability of the equipment were achieved. A supervised OPLS-DA was conducted to maximize the separation and mine differential metabolites, and better separations were achieved (Figure S2B, F). The Q2 and R2Y values of OPLS-DA positive model were 0.981 and 0.931, accordingly. And the Q2 and R2Y values of negative model were 0.976 and 0.933, respectively, demonstrating favorable models with good fitness and predictive ability (Figure S2 C, G). In addition, permutation tests with 200 iterations were used to assess the possibility of model over-fitting. While both the permuted *R*2 and *Q*2 values in positive and negative models were more than 0.95, *p* values were all less than 0.05, suggesting valid models(Figure S2 D, H).

### Immunoprecipitation (IP) and western blot (WB) analysis

Cells were lysed with cell lysis buffer (abcam, UK) or RIPA(NCM Biotech, China) consisting of phosphatase and protease inhibitor mix (NCM Biotech, China). To prepare the protein samples for immunoprecipitation (IP), protein complexes were precipitated from whole-cell lysis according to the specifications with IgG control or indicated antibodies before precipitation with protein A/G beads (MCE, USA). To evaluate the expression levels of certain protein, protein complexes or the protein samples for immunoprecipitation(IP) were separated via 10% SDS-PAGE then transferred onto PVDF membranes (Millipore, USA). The membranes were blocked with quick blocking buffer(NCM, China) for 10 min and incubated with primary antibody overnight at 4 °C. After washed with TBST (20 mM Tris-base, 135 mM NaCl, and 0.1% Tween 20), the membranes were incubated with secondary antibody for 1 h at room temperature. The signals were detected in an imaging system by using enhanced chemiluminescence kit. Relative of expressions protein were determined by ImageJ normalized by *β-*actin.

### Fluorescent quantitative real-time PCR (RT-qPCR)

Total RNA were extracted with TRIzol (Invitrogen, USA). The HyperScript III 1st Strand cDNA Synthesis Kit with gDNA Remover (NovaBio, China) were applied to synthesize the cDNA according to the manufacturer's instructions. RT-qPCR was performed in 20 μL reactions using 2×S6 Universal SYBR qPCR Mix (NovaBio, China) on a Real-Time PCR Systems (Thermo Scientific). Comparison of 2^−ΔΔCt^ were used to assess the expression levels of certain gene after being normalized by β-actin. All experiments were performed triplicate. Primer of sequence for RT-qPCR of mRNAs were shown in Table S2.

### Plasmids, transfection, and cell screening

DNA plasmids were transfected into cells using Lipofectamine 3000 (Fisher Scientific). Lentivirus for infection of target cell lines were prepared with pCMV dR8.9 and pCMV-VSV-G plasmids by HEK293T cells. Then cells were infected by specific lentivirus and screened by puromycin. Overexpression and knockdown efficiencies were verified by RT-qPCR and WB assays. The information of plasmids and oligos used in the study were listed in Table S3.

### Intracellular NADPH, NADPH/NADP+ ratio, GSH, GSH/GSSG ratio, ROS level, and G6PD activity measurement

The intracellular NADPH level and NADPH/NADP^+^ ratio were determined using a NADPH/NADP^+^ Quantitation Kit (Beyotime, China) and NADP/NADPH-Glo Assay (Promega, USA) according to the manufacturer’s standard protocol. The intracellular GSH level and GSH/GSSG ratio were detected by GSH and GSSG Assay Kit (Beyotime, China) and GSH/GSSG Ratio Detection Assay Kit II (Fluorometric-Green) (abcam, UK) following the manufacturer’s instructions. ROS level was evaluated by a reactive oxygen species assay kit (Beyotime, China) according to the manufacturer’s standard protocol. The G6PD enzyme activity was measured using glucose-6-phosphate dehydrogenase assay kit (Colorimetric)(abcam, UK) following the manufacturer’s instructions.

### Data analysis

GraphPad Prism 8.0 and SPSS 25.0 were used for statistical analysis. All quantitative data are presented as the mean ± SD of at least three independent experiments. *P* values were determined by *t* test or one-way analysis of variance (ANOVA).

## Result

### Altered phenotype in regorafenib-resistant cells

**Fig. 1:**
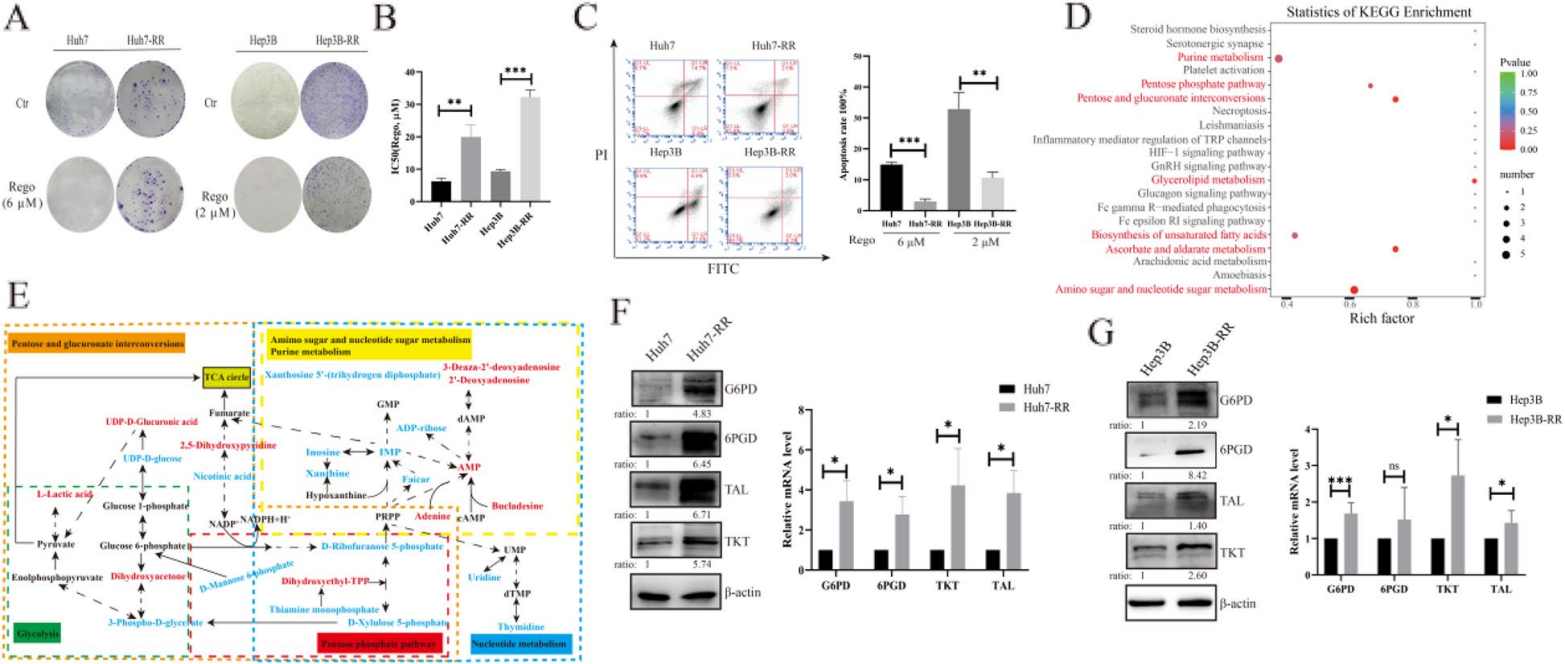
Upregulated PPP in regorafenib-resistant cells. **A** Colony formations were detected on drug-sensitive and regorafenib-resistant cells and cells were treated with or without regorafenib for 48 h, *n* = 3. **B** Half maximal inhibitory concentration (IC_50_) of regorafenib performed on drug-sensitive and regorafenib-resistant cells and cells were treated with gradient concentration of regorafenib for 24 h, *n* = 3. **C** Apoptosis plot diagram(left) and apoptosis rate(right) were detected by FCM and cells were treated with regorafenib for 24 h. **D** The differential metabolites that displayed significantly different abundances between drug-sensitive (Huh7) and regorafenib-resistant cells (Huh7-RR) were subjected for KEGG analysis. The vertical axis represents top metabolic pathways and the horizontal axis represents the importance value of pathways. *n* = 8. **E** The metabolic network of potential biomarkers. Upregulated substances were colored with red; downregulated substances were colored with blue; undetected substances were colored with black. IMP, Hypoxanthine-9-β-D-Arabinofuranoside; AMP, Adenosine monophosphate; PRPP, Phosphoribosyl pyrophosphate; UMP, Uridine monophosphate; dTMP, deoxythymidine monophosphate. **F** Protein (left, *n* = 3) and mRNA (right, *n* = 3) expression of PPP in Huh7 and Huh7-RR. **G** Protein (left, *n* = 3) and mRNA (right, *n* = 5) expression of PPP in Hep3B and Hep3B-RR. ^*^P < 0.05, ^**^P < 0.01, ^***^P < 0.001, mean ±SD

First, the intermittent and gradual increase of dose therapy was used to construct HCC regorafenib-resistant cell lines, which were named Huh7-RR and Hep3B-RR.The drug resistant characteristics of regorafenib-resistant cells were evaluated by half maximal inhibitory concentration (IC_50_), colony formation and cell apoptosis rate. Compared with drug-sensitive cells, increased colony formations were displayed in regorafenib-resistant cells whether treated with or without regorafenib (Fig. 1A), demonstrating better cell proliferation in regorafenib-resistant cell lines. In addition, increased IC_50_ of regorafenib was displayed in regorafenib-resistant cells (Fig. 1B). Moreover, the apoptosis rates of regorafenib-resistant cells were significantly decreased (Fig. 1C). These results indicated that regorafenib-resistant cells were less sensitive to regorafenib. Therefore, stable resistant phenotypes of resistant cells were achieved, demonstrating reliable regorafenib-resistant cell lines in HCC were obtained.

### Altered metabolic profiling of regorafenib-resistant cells

To investigate the unknown metabolic alteration in regorafenib-resistant cells, an untargeted metabolomic analysis were performed between drug-sensitive cells and regorafenib-resistant cells. To obtain the metabolic profiling, we firstly performed an unsupervised PCA. As the PCA score plots displayed, there was a remarkable separation between Huh7 group and Huh7-RR group (Figure S2A, E), indicating that the metabolic profile of regorafenib-resistant cells was completely different from drug-sensitive HCC cells. Statistical analysis of the LC-MS data by volcano plot and heat map of cluster analysis(Figure S3A, B) showed significant metabolic differences between drug-sensitive and regorafenib-resistant cells. The Kyoto encyclopedia of genes and genomes (KEGG, http://www.genome.jp/kegg/) is a powerful database, which could excavate potential pathways. Therefore, KEGG analysis of differential metabolites was performed to explore the altered metabolic pathways in regorafenib-resistant cells. KEGG analysis demonstrated that these metabolites were enriched from pentose phosphate pathway (PPP), pentose and glucuronate interconversions, purine metabolism, amino sugar and nucleotide sugar metabolism, biosynthesis of unsaturated fatty acids and glycerolipid metabolism etc. (Fig. 1D). The perturbed metabolites associated with the major pathways in regorafenib-resistant cells were shown in Table S4. The heat map of cluster analysis showed that the intermediate metabolites of the pentose phosphate pathway, including D-ribofuranose-5-phosphate (R5P), D-xylulose-5-phosphate, 3-phospho-D-glycerate, and D-mannose-6-phosphate were significantly decreased (Figure S4, blue rectangle), while nucleic acid synthetic intermediates were accumulated, including 3-deaza-2′-deoxyadenosine, 2′-deoxyadenosine, bucladesine, UDP-D-glucuronic acid, and AMP (Figure S4, orange rectangle). To elucidate the relationship of related metabolites from main pathways, metabolite networks were delineated based on KEGG and information of website HMDB. These major metabolites were mainly from glucose catabolism and nucleotide metabolism (Fig. 1E). PPP, a branch of carbohydrate metabolism, produces NADPH and R5P in the oxidative phase and turn carbons such as glycerol-3-phosphate back to the glycolytic or gluconeogenic pathway in the nonoxidative phase [16]. It has been reported that nicotinamide adenine dinucleotide phosphate (NADPH) is involved in fatty acid synthesis and the conversion of R5P into phosphoribosyl pyrophosphate (PRPP) is involved in the de novo synthesis and salvage synthesis of nucleic acids [17]. Interestingly, the interconversion of pentose and glucuronic acid, amino sugar and nucleotide sugar metabolism, and purine metabolism are linked by PPP. Therefore, glycolysis and nucleotide metabolism may play a vital part in regorafenib resistance in HCC. Foremost, PPP may be the most relevant pathway regulating regorafenib resistance in HCC.

### Upregulated PPP in regorafenib-resistant cells

PPP is an alternative pathway of glucose catabolism, which is mainly regulated by glucose-6-phosphate dehydrogenase(G6PD), 6-phosphogluconate dehydrogenase(6PGD), transketolase (TKT), and transaldolase 1(TAL), etc. Metabolomic analysis suggested that PPP may be the most relevant pathway regulating regorafenib resistance in HCC, but its alteration was not validated. Therefore, the protein and mRNA levels of G6PD, 6PGD, TAL, and TKT were detected to evaluate the perturbation of PPP. Compared with Huh7 cells, the protein and mRNA expressions of G6PD, 6PGD, TAL, and TKT in Huh7-RR cells were significantly increased (Fig. 1F). Similar trend was observed in Hep3B-RR cells (Fig. 1G). These results demonstrated that the enzymes related to PPP were significantly increased in both protein and mRNA levels in regorafenib-resistant cells, suggesting that PPP metabolism was up-regulated in regorafenib-resistant cells.

### G6PD inhibition or deletion enhances the effect of regorafenib on regorafenib-resistant cells in HCC

**Fig. 2:**
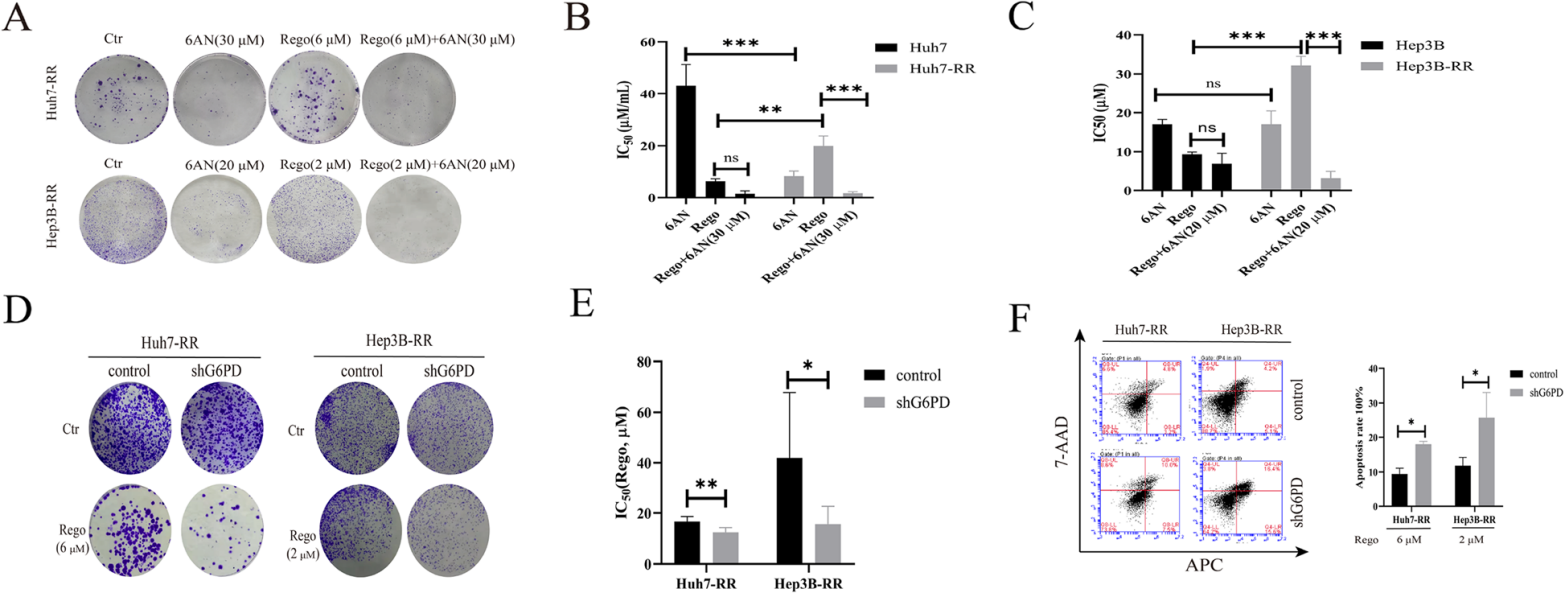
G6PD inhibition or deletion enhances the effect of regorafenib on HCC regorafenib-resistant cells. **A** Colony formations were detected on drug-sensitive and regorafenib-resistant cells which were treated with or without regorafenib, 6AN or the combination of regorafenib and 6AN for 48 h, *n* = 3. **B, C** Half maximal inhibitory concentration (IC_50_) of regorafenib, 6AN or the combination of regorafenib and 6AN were performed on drug-sensitive and regorafenib-resistant cells and cells were treated with gradient concentration of regorafenib, 6AN or the combination of regorafenib and specific 6AN for 24 h, *n* = 3. **D** Effect of G6PD knockdown on regorafenib-resistant cell sensitivity to regorafenib was determined by colony formations and cells were treated with or without regorafenib for 48 h, *n* = 3. **E** IC_50_ of regorafenib were performed on G6PD knockdown on regorafenib-resistant cell and cells were treated with gradient concentration of regorafenib for 24 h, *n* = 3. **F** Apoptosis plot diagram (left) and apoptosis rate (right) were detected by FCM and cells were treated with regorafenib for 24 h, *n* = 3. ^*^P < 0.05, ^**^P < 0.01, ^***^P < 0.001, mean ±SD

G6PD is the key enzyme of PPP, we focused on G6PD to further explore the effects of PPP on regorafenib-resistance in HCC. 6-aminonicotinamide (6AN), a G6PD inhibitor, was used to investigate regorafenib-resistance in HCC. First, 6AN could impairs the colony formation (Fig. 2A) and cell viability (Figure S5A) of regorafenib-resistant cells. The combination of regorafenib and 6AN decreased the colony formation (Fig. 2A) and cell viability(Figure S5A) of regorafenib-resistant cells, and IC_50_ (Fig. 2B) of regorafenib on regorafenib-resistant cells. Similar to the effects of 6AN, G6PD ablation decreased the colony formation (Fig. 2C) and IC_50_ (Fig. 2D) of regorafenib on regorafenib-resistant cells. In addition, increased cell apoptotic rates to regorafenib has been observed in G6PD knocked-down regorafenib-resistant cells (Fig. 2E). These results demonstrated that G6PD inhibition or deletion could retard the growth of cells and enhance the effect of regorafenib on regorafenib-resistant cells in HCC. Therefore, targeting G6PD could reverse regorafenib-resistance in HCC.

### G6PD overexpression impairs the effect of regorafenib on HCC

**Fig. 3:**
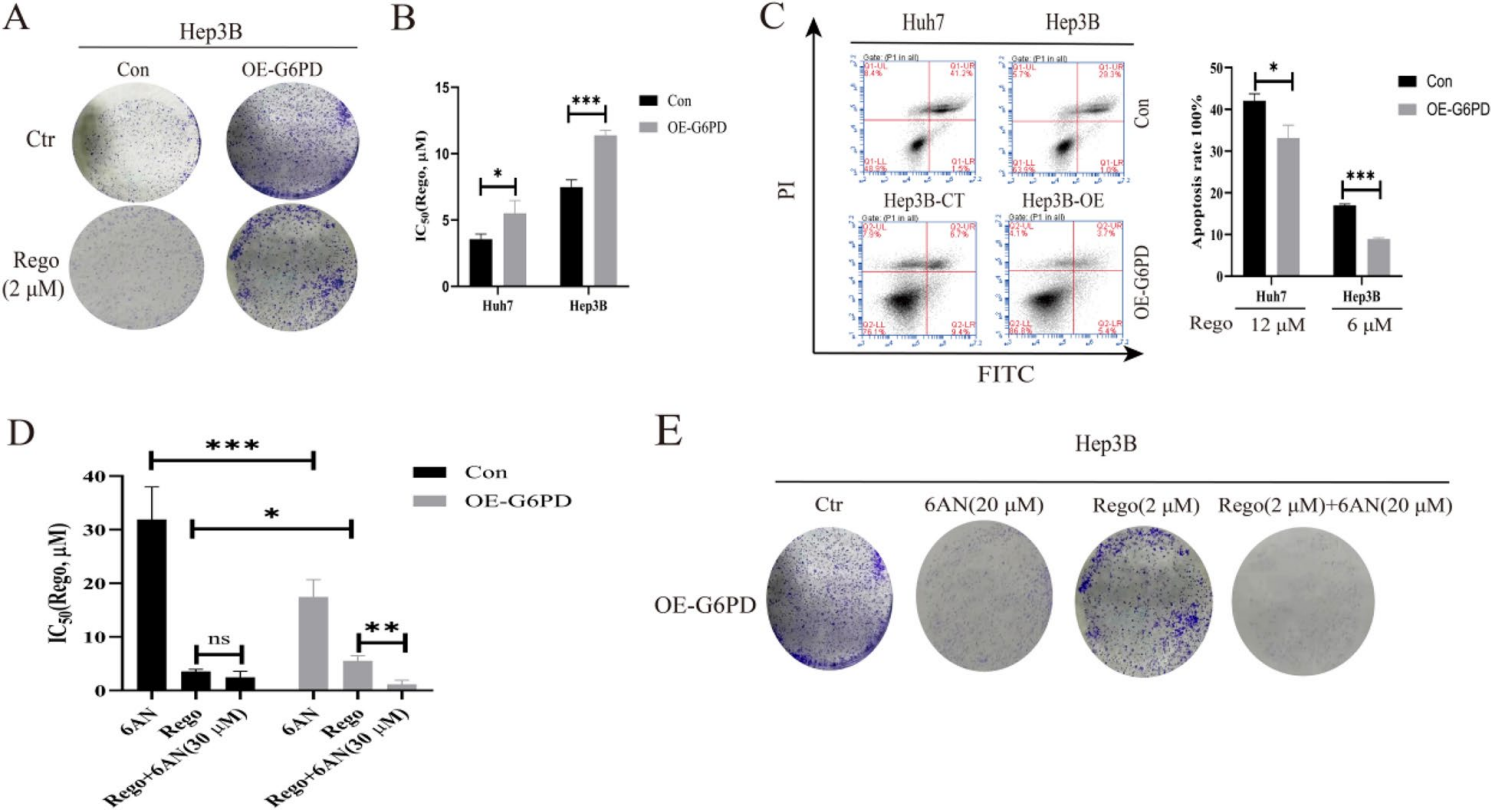
G6PD overexpression impairs the effect of regorafenib on HCC. **A** Effect of G6PD overexpression on drug-sensitive cell sensitivity to regorafenib was determined by colony formations and cells were treated with or without regorafenib for 48 h, *n* = 3 (Hep3B cell line). **B** IC_50_ of regorafenib were performed on G6PD overexpressed cells and its counterparts and cells were treated with gradient concentration of regorafenib for 24 h, *n* = 3. C. Apoptosis plot diagram (left) and apoptosis rate(right) were detected by FCM and cells were treated with regorafenib for 24 h. *n* = 3. **D** IC_50_ of regorafenib, 6AN or the combination of regorafenib and 6AN were performed on drug-sensitive and G6PD overexpression in drug-sensitive cells and cells for 24 h, *n* = 3 (Huh7 cell line). **E** Colony formations were detected on drug-sensitive and regorafenib-resistant cells which were treated with or without regorafenib, 6AN, or the combination of regorafenib and 6AN for 48 h, *n* = 3 (Hep3B cell line). ^*^P < 0.05, ^**^P < 0.01, ^***^P < 0.001, mean ±SD

In order to further investigate the effects of G6PD on regorafenib-resistance, we established G6PD overexpressed cell lines of HCC. We found that G6PD overexpressed cell lines of HCC had similar phenotype with regorafenib-resistant cell lines. The colony formations were increased in G6PD overexpressed cell lines whether treated with or without regorafenib (Fig. 3A). The results were in accord with the effects of high G6PD expression could underpin cell growth reported by Zhang Y et al. [18]. In addition, G6PD overexpressed cell lines displayed increased IC_50_ of regorafenib (Fig. 3B) and decreased cell apoptotic rates under regorafenib treatment (Fig. 3C). Moreover, the combination of regorafenib and 6AN decreased the colony formation (Fig. 3E) , cell viability (Figure S5B) and IC_50_ (Fig. 3D) of regorafenib in G6PD overexpressed cells of HCC, which demonstrated that 6AN removed the effect of G6PD overexpression on regorafenib resistance. Altogether, G6PD overexpression could underpin cell growth and impairs the effect of regorafenib to HCC.

### Enhanced PPP increased the anti-oxidative stress capabilities of regorafenib-resistant cells

**Fig. 4:**
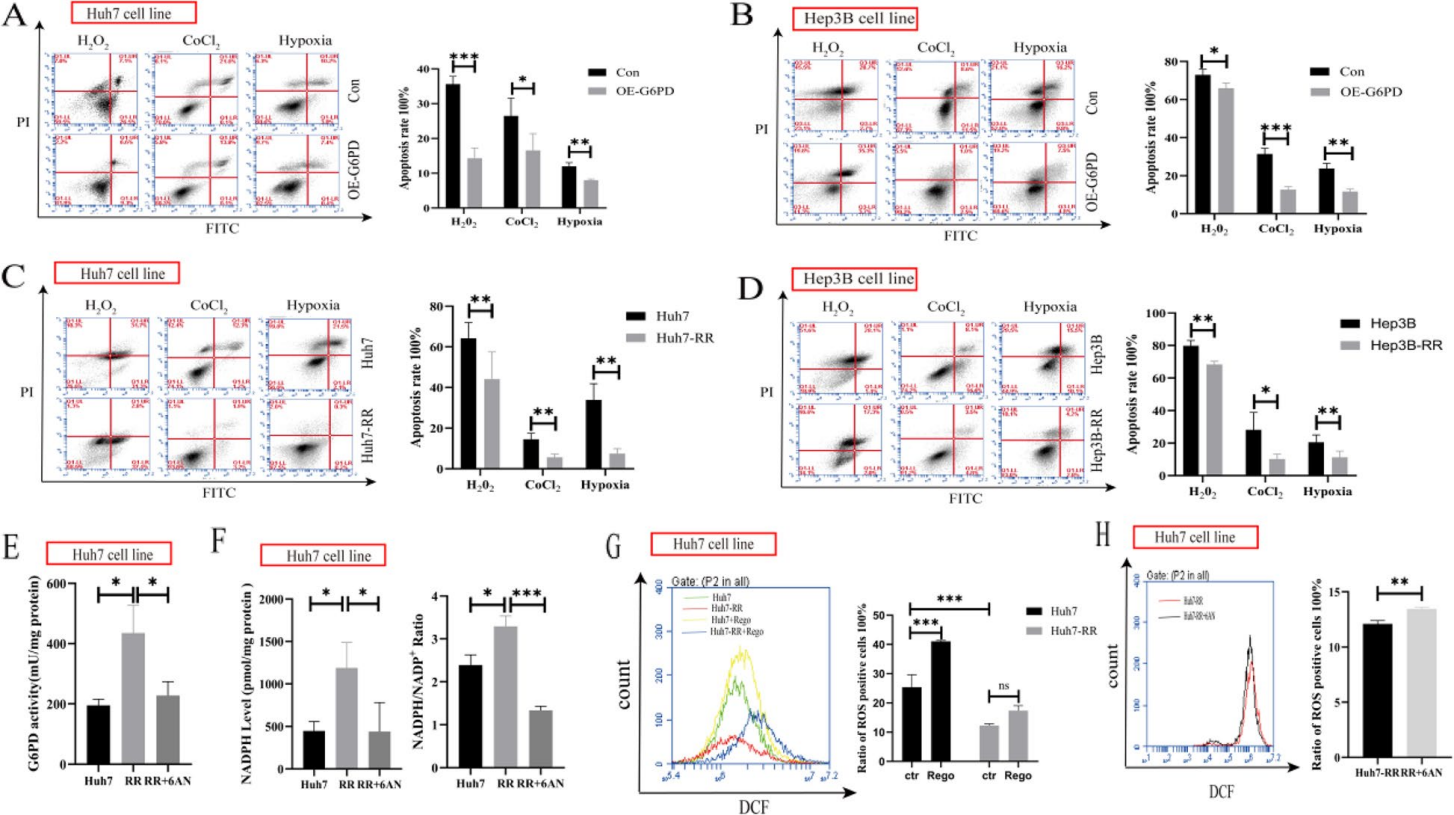
Enhanced PPP increased the anti-oxidative stress capabilities of regorafenib-resistant cells. **A**, **B** The effects of G6PD overexpression on cell apoptosis rate of drug-sensitive HCC cells under H_2_O_2_ (100 μM), CoCl_2_ (100 μM), and hypoxia, *n* = 3. **C**, **D** Cell apoptosis rate under H_2_O_2_ (100 μM), CoCl_2_ (100 μM) and hypoxia were detected in drug-sensitive and regorafenib-resistant cells, *n* = 3. **E** G6PD enzymatic activity were recorded in drug-sensitive and regorafenib-resistant cells treated with or without 6AN (30 μM), * n *= 3 (Huh7 cell line). **F** NADPH level (left) and NADPH/NADP+ ratio (right) were detected in drug-sensitive and regorafenib-resistant cells treated with or without 6AN (30 μM), * n *= 3 (Huh7 cell line). **G** ROS-positive cells (left) and ratio to total cells (right) were analyzed in drug-sensitive and regorafenib-resistant cells treated with or without regorafenib, n=3(Huh7 cell line). **H** The number of ROS-positive cells (left) and the ratio to the total number of cells (right) were analyzed in regorafenib-resistant cells treated with or without 6AN (30 μM), *n* = 3 (Huh7 cell line). ^*^P < 0.05, ^**^P < 0.01, ^***^P < 0.001, mean ±SD

Our study has revealed that PPP was enhanced in regorafenib-resistant cells and we speculated that the enhanced PPP may be a pivotal factor for regorafenib-resistant cells to suffer regorafenib stimulation. In a variety of cancers, G6PD is essential for maintaining low levels of reactive oxygen species(ROS) and reduced glutathione/oxidized glutathione ratio(GSH/GSSG) balance in that G6PD creates NADPH to maintain redox balance and promotes tumor cell proliferation and inhibits cell apoptosis [19]. Firstly, we explored the anti-oxidative stress ability of regorafenib-resistant cells. Under the conditions of H_2_O_2_, CoCl_2_, and hypoxia, G6PD overexpression reduced cell apoptosis levels of HCC cells (Fig. 4A, B), indicating that G6PD overexpression increased the anti-oxidative stress ability of HCC cells. Similar to G6PD overexpressed HCC cells, cell apoptosis rates of regorafenib-resistant cells were decreased under conditions of H_2_O_2_, CoCl_2_ and hypoxia when compared with HCC cells (Fig. 4C, D), demonstrating that anti-oxidative stress ability was increased in regorafenib-resistant cells, which may depend on increased G6PD expression. Moreover, compared with Huh7 cells, G6PD enzymatic activity (Fig. 4E), NADPH levels and NADPH/NADP^+^ ratio (Fig. 4F) significantly increased in Huh7-RR cells, suggesting enhanced PPP in Huh7-RR cells. Interestingly, ROS level increased by regorafenib in the Huh7 cells (Fig. 4G), while there was no significant change in the Huh7-RR cells. In addition, ROS level in the Huh7-RR cells was significantly lower than that in the Huh7 cells (Fig. 4G), indicating that the anti-oxidative capacity of regorafenib-resistant cells was better than that of Huh7 cells, which could clean ROS induced by regorafenib. However, G6PD inhibitor decreased the G6PD enzymatic activity (Fig. 4E), NADPH and NADPH/NADP^+^ ratio (Fig. 4F), but increased ROS level of Huh7-RR cells (Fig. 4H). These results indicated that the increased anti-oxidative stress capacity of Huh7-RR was regulated by G6PD. Therefore, enhanced PPP enabled regorafenib-resistant cells better capacity to balance cellular oxidative stress, which could clear ROS induced by regorafenib.

### A feedback loop of PPP and PI3K/AKT signal pathway drives HCC regorafenib-resistance

**Fig. 5:**
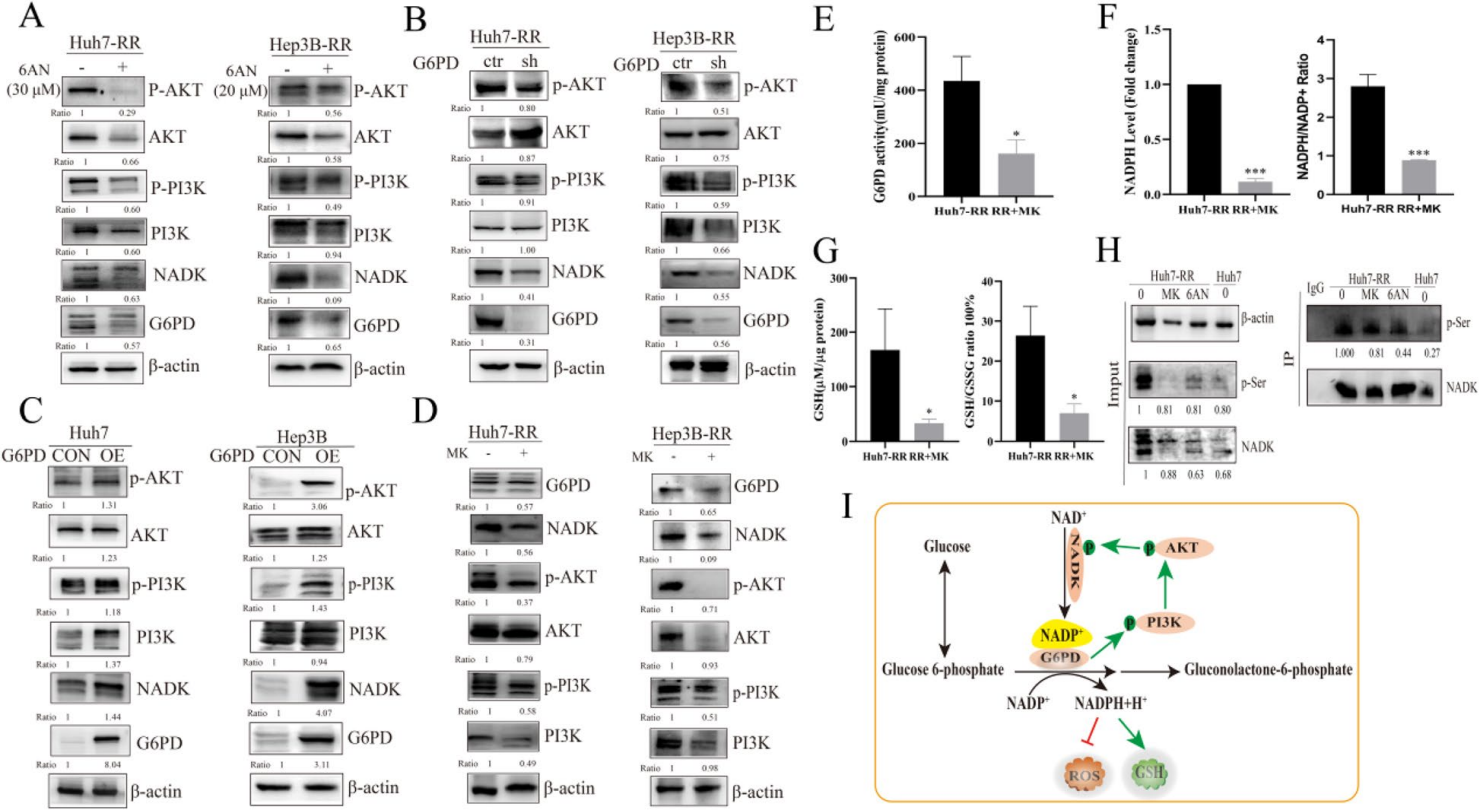
A feedback loop of PPP and PI3K/AKT signal pathway drives regorafenib-resistance in HCC. **A** The effects of G6PD inhibition by 6AN on the expression of NADK and activation of PI3K/AKT signal pathway in drug-resistant cells, *n* = 4. **B** The effects of G6PD knockdown on the expression of NADK and activation of PI3K/AKT signal pathway in drug-resistant cells, *n* = 3. **C** The effects of G6PD overexpression on the expression of NADK and activation of PI3K/AKT signal pathway in drug-sensitive cells, *n* = 3. D. The effects of PI3K/AKT signal pathway inhibition by MK-2206(MK, 10 μM) on the expression of G6PD and NADK in drug-sensitive cells, *n* = 3, *n* = 3. **E** The effects of PI3K/AKT signal pathway inhibition by MK (10 μM) on G6PD enzymatic activity in regorafenib-resistant cells, *n* = 3 (Huh7 cell line). **F** The effects of PI3K/AKT signal pathway inhibition by MK (10 μM) on NADPH level (left) and NADPH/NADP^+^ ratio (right) in drug-resistant cells, *n* = 3 (Huh7 cell line). **G** The effects of PI3K/AKT signal pathway inhibition by MK(10 μM) on GSH level (left) and GSH/GSSG ratio (right) in drug-resistant cells, *n* = 3 (Huh7 cell line). **H** The effects of PI3K/AKT signal pathway by MK (10 μM) or G6PD inhibition by 6AN (30 μM) on phosphorylation level of NADK were detected by immunoprecipitation in drug-sensitive and regorafenib-resistant cells, *n* = 1 (Huh7 cell line). **I** A schematic model of a feedback loop of PPP and PI3K/AKT signaling pathway inducing regorafenib-resistance in HCC.^*^P < 0.05, ^**^P < 0.01, ^***^P < 0.001, mean ±SD

PI3K/AKT signaling pathway is a classic signaling pathway for tumor development and drug resistance. The combination of PI3K/AKT signaling pathway inhibitor and regorafenib could enhance the curative effect of regorafenib on HCC by inhibiting the proliferation and promoting apoptosis of HCC cells [20, 21]. In order to clarify the mechanism of PPP regulating regorafenib resistance, we investigated the role of PI3K/AKT signaling pathway in regorafenib resistance. The protein levels of PI3K, p-PI3K, AKT, and p-AKT and the ratio of p-PI3K/PI3K and p-AKT/AKT were significantly increased in regorafenib-resistant cells (Figure S6A), indicating that the PI3K/AKT signaling pathway was activated and may be related to regorafenib-resistance. Interestingly, PI3K/AKT signaling pathway was inhibited under the condition of G6PD inhibition or deletion (Fig. 5A, B), but activated while G6PD was overexpressed (Fig. 5C) (the results was in line with our previous studies), which demonstrating that PI3K/AKT signaling pathway was modulated by G6PD. In addition, the protein (Fig. 5D) and mRNA (Figure S6C) expression and enzymatic activity (Fig. 5E) of G6PD were decreased in regorafenib-resistant cells when treated with MK-2206 (MK), an inhibitor of PI3K/AKT signaling pathway. These results was consistent with previous studies that protein expression [22, 23] and enzyme activity [24] of G6PD were regulated by PI3K/AKT signaling pathway performed on other kinds of cancer cells. The level of NADPH and the ratio of NADPH/NADP^+^ (Fig. 5F), GSH and ratio of GSH/GSSG (Fig. 5G) were declined by MK as well. These results proved that G6PD was regulated by PI3K/AKT signaling pathway. Therefore, a feedback loop of PPP and PI3K/AKT signal pathway may drive regorafenib resistance in HCC.

In mammalian cells, NADP^+^, which is converted from NAD^+^ under the catalysis of NADK, is a coenzyme of G6PD and is able to increase the stability and activity of G6PD [25]. In this study, the protein (Figure S6A) and mRNA (Figure S6B) expression of NADK increased in regorafenib-resistant cells, indicating that NADK probably mediates regorafenib resistance. Similar to PI3K/AKT signaling pathway, NADK was declined under the condition of G6PD inhibition or deletion (Fig. 5A, B), but increased while G6PD was overexpressed (Fig. 5C), which demonstrating that NADK was modulated by G6PD. In addition, the protein (Fig. 5D) and mRNA (Fig. S6C) NADK was decreased in regorafenib-resistant cells when treated with MK, which proved that NADK was regulated by PI3K/AKT signaling pathway. The activation of PI3K/AKT signaling pathway could increase the enzymatic activity of NADK and promote the generation of NADP^+^ through increasing the phosphorylation of NADK [26]. Therefore, we speculated that PI3K/AKT signaling pathway possibly regulated G6PD activity by modulating NADK to affect the synthesis of NADP^+^. Next, a universal serine phosphorylation antibody was applied to investigate the enzymatic activity of NADK by detecting the phosphorylation level of NADK. Compared with drug-sensitive cells, NADK increased in regorafenib-resistant cells, but decreased when treated with MK or 6AN (Fig. 5H, left), which was consistent with previous study described above. Compared with Huh7 cells, the level of serine phosphorylation of NADK in Huh7-RR cells increased in the extracted immunoprecipitation, but declined when treated with MK or 6AN (Fig. 5H, right). These results showed that NADK activity in regorafenib-resistant cells was increased, but decreased both when PI3K/AKT and G6PD inhibition, suggesting G6PD possibly regulated the enzymatic activity and protein expression of NADK by controlling PI3K/AKT signaling pathway.

## Discussion

Currently, HCC ranks as a leading fatal malignant tumor with rapidly increasing incidence rate and low 5-year survival rate (20%) worldwide [27]. As a strategy for second line therapy for HCC, regorafenib remains the mainstay for improving the prognosis of sorafenib-resistant patients in HCC. To our knowledge, metabolic profiling of regorafenib-resistant cells remains largely unknown. This is the first study revealed that PPP may was a key metabolic pathway regulating regorafenib resistance based on untargeted metabolomics. In recent years, it has been reported that PPP promotes tumorigenesis and drug resistance. The inhibition or knockdown metabolic enzymes and reduction of enzyme activity of PPP are capable to induce cell apoptosis and reduce cell proliferation, which reversed drug resistance [28, 29]. But the role of PPP in regorafenib resistance in HCC is still unknown. Our results showed that both the protein and mRNA expressions of G6PD, 6PGD, TAL, and TKT in regorafenib-resistant cells were significantly increased, indicating that PPP was enhanced in regorafenib-resistant cells.

As a key enzyme of PPP, G6PD plays a pivotal role in the development of tumor [30] and the occurrence of drug resistance in various tumors [31–33]. However, the role of G6PD in regorafenib resistance is still unrevealed. In this study, G6PD inhibition or knockdown increased the sensitivity of regorafenib-resistant cells to regorafenib, but G6PD overexpression reduced the sensitivity of drug-sensitive cells to regorafenib, indicating that G6PD derived regorafenib resistance in HCC. The endurance capability for oxidative stress of regorafenib-resistant cells was mimic to sensitive cells that overexpressed G6PD while compared with their counterparts respectively. Moreover, increased G6PD enzyme activity, NADPH and NADPH/NADP^+^ and decreased ROS level were observed in regorafenib-resistant cells, but these effects were eliminated by G6PD inhibition in regorafenib-resistant cells. These results demonstrated that regorafenib-resistant cells adapted to regorafenib by enhancing PPP.

PI3K/AKT is a typical signaling pathway associated in tumorigenesis and drug resistance [34, 35]. In this study, PI3K/AKT signaling pathway was activated in regorafenib-resistant cells, indicating that PI3K/AKT signaling pathway mediated regorafenib resistance in HCC. We found that G6PD inhibition or knockdown in regorafenib-resistant cells inhibited the activity of PI3K/AKT signaling pathway. On the contrary, G6PD overexpression further activated PI3K/AKT signaling pathway. These results demonstrated that G6PD induced regorafenib resistance in HCC by activating PI3K/AKT signaling pathway. Interestingly, the inhibition of PI3K/AKT signaling pathway decreased the expression and enzyme activity of G6PD, and the ratio of NADPH/NADP^+^ and GSH/GSSG, indicating that G6PD was regulated by PI3K/AKT signaling as well. Therefore, a feedback loop of PPP and PI3K/AKT signaling pathway was associated with regorafenib resistance.

Previous study has revealed that NADK knockdown or inhibition inhibited tumor proliferation [36].We found that the expression of NADK was increased in regorafenib-resistant cells. Inspired by the report that NADK enzyme activities were positively regulated by PI3K/AKT signaling pathway [26], we speculated that NADK may play a key role in the loop of PI3K/AKT signaling pathway and PPP. In this study, the expression and enzyme activity of NADK were both positively regulated by G6PD and PI3K/AKT signaling pathway. These results suggested that G6PD-PI3K/AKT-NADK-NADP+ axis mediated with regorafenib resistance in HCC.

## Conclusion

Our novel findings revealed that HCC cells resistant to regorafenib have upregulated PPP. G6PD is a promising target to reserve regorafenib-resistance in HCC. Enhanced PPP metabolism allowed regorafenib-resistant cells to have a better capacity to adapt to oxidative stress caused by regorafenib. G6PD-PI3K/AKT-NADK-NADP^+^ may form a loop to regulate regorafenib resistance in HCC (Fig. 5I). Overall, the activation of this feedback loop can maintain the balance of cellular oxidative stress, promoting cell proliferation and reducing apoptosis, which lead to regorafenib resistance in HCC.

### Supplementary Information

**Additional file 1:** **Table S1.**The key chemicals andmaterials used in this study. **Table S2. **Primer of sequence for RT-qPCR ofmRNAs. **Table S3.** Plasmids and oligosused in the study.

**Additional file 2:** **Table S4.** Identification of primary metabolites.

**Additional file 3:** **Figure S1.** Multivariate statistical analysis and metabolic profiling. (A) total ion chromatogram(TIC) of QCsamples. (B) PCA analysis of QC. **Figure S2.** Multivariate modelling of LC-MS data. (A) PCA score plot in positivemode. (B) OPLS-DA score plot in positive mode. (C) Validation of positive mode.(D) Permutation tests of positive mode. (E) PCA score plot in negative mode. (F)OPLS-DA score plot in negative mode. (G) Validation of negative mode. (H) Permutationtests of negative mode. **Figure S3.** Differentialmetabolites variation. (A) The volcano plots(red spots represents increasedmetabolites, red spots represents decreased metabolites and gray spotsrepresents not significant metabolites); (B) cluster analysis of heat map(colorred represents increased metabolites, and the color green represents decreased metabolites).The selection of differential metabolites was according to variable importancein projection(VIP) based on OPLS-DA, *p*value fromStudent’s t-test and fold change. Peaks with VIP≥1, *P*<0.05，foldchange≥ 2 and fold change≤ 0.5 were included in as differential metabolites. **Figure S4.** Heat map of cluster analysisof potential biomarkers. Color red represents increased metabolites, and thecolor green represents decreased metabolites. **Figure S5.** G6PD inhibition the effect of regorafenib onregorafenib-resistant cells in HCC. (A) Cellviability were detected on Huh7 and Huh7-RR cells which were treated with or withoutregorafenib(6 μM), 6AN(30 μM) or the combination of regorafenib(6 μM) and 6AN(30 μM) for 48 h, *n*=3. (B) Cellviability were detected on G6PD overexpressed cells and it’s counterparts andcells which were treated with or without regorafenib(6 μM), 6AN(30 μM) or thecombination of regorafenib(6 μM) and 6AN(30 μM) for 48 h, *n*=3. **Figure S6.** PI3K/AKTsignaling pathway and NADK were involved in the mechanism of G6PD inducedregorafenib-resistance in HCC. (A) Protein levels of PI3K(PI3Kinase p85), p-PI3K(Phospho-PI3-kinase p85-α/γ(Tyr467/199)), AKT(pan),p-AKT(ser473) and NADK were detected by western blot in Huh7 and Huh7-RR (left, *n*=3),Hep3B and Hep3B-RR(right, *n*=3). (B)The mRNA levels of NADK, *n*=3. (C) Theeffects of PI3K/AKT signal pathway inhibition by MK-2206(MK, 10 μM) on the mRNA levels of G6PD and NADK in regorafenib-resistant cells, *n*=3.

## Data Availability

The used datasets are available from the corresponding author upon reasonable request.

## Abbreviations

dTMP: deoxythymidine monophosphate
HCC: Hepatocellular carcinoma
IC_50_: Half maximal inhibitory concentration
IMP: Inosine 5′-monophosphate
GMP: Guanosine 5′-monophosphate
R5P: Ribose-5-phosphate
PPP: Pentose phosphate pathway
PRPP: Phosphoribosyl pyrophosphate
NADPH: Nicotinamide adenine dinucleotide phosphate
G6PD: Glucose-6-phosphate dehydrogenase
6PGD: 6-phosphogluconate dehydrogenase
TKT: Transketolase
TAL: Transaldolase 1
NADK: Nicotinamide adenine dinucleotide kinase
6AN: 6-Aminonicotinamide
ROS: Reactive oxygen species
GSH/GSSG: Reduced glutathione/oxidized glutathione ratio
MK: MK-2206
UMP: Uridine monophosphate
