# Supplementary material for: A feedback loop of PPP and PI3K/AKT signal pathway drives regorafenib-resistance in HCC
Source: Cancer Metab. 2023 Dec 18;11:27. doi: 10.1186/s40170-023-00311-5 (PMC10726576; doi:10.1186/s40170-023-00311-5)
Supplement: Supplementary file 1 — Additional file 1: Table S1.The key chemicals andmaterials used in this study. Table S2. Primer of sequence for RT-qPCR ofmRNAs. Table S3. Plasmids and oligosused in the study. [file 40170_2023_311_MOESM1_ESM.doc]

Supplementary Information

A feedback loop of PPP and PI3K/AKT signal pathway drivers regorafenib-resistance in HCC

Huihua Yang^1,2†^, Dahong Chen^1†^, Yafei Wu^1^, Heming Zhou^1^, Wenjing Diao^1^, Gaolin Liu^1*^, Qin Li^1*^

Table S1 The key chemicals and materials used in this study.

| **Chemicals or materials** | **Source** | **Identifier** |
| --- | --- | --- |
| **Antibodies** |  |  |
| β-actin mouse mAb | CST | 58169S |
| G6PD Rabbit mAb | CST | 12263S |
| Anti-PGD Rabbit PolyAb | Absin | abs140793 |
| Anti-TAL Rabbit PolyAb | Absin | abs110676 |
| Anti-TKT Rabbit PolyAb | Absin | abs117073 |
| NADK Rabbit mAb | CST | 55948S |
| p-Ser Antibody (16B4) | SantaCruz | sc-81514_SAMPLE |
| Akt (pan) Rabbit mAb | CST | 4685S |
| Phospho-Akt(Ser473) Rabbit mAb | CST | 4060S |
| Rabbit (DAE1) mAb IgG XP（R） Isotype Control | CST | 3900S |
| PI3 Kinase p85 Rabbit mAb | CST | 4257S |
| Phospho-PI3-kinase p85-α/γ(Tyr467/199) Rabbit PolyAb | Absin | abs130869 |
| Goat Anti-rabbit IgG-HRP | Absin | abs20040ss |
| Goat Anti-mouse IgG-HRP | Absin | abs20039ss |
| **Chemicals and Inhibitors** |  |  |
| regorafenib | MCE | HY-10331 |
| 6-aminonicotinamide | MCE | 87547 |
| CoCl_2_ · 6H_2_O | Sigma-Aldrich | C8661-25G |
| H_2_0_2_ | Amresco | E882 |
| puromycin | MCE | 87126 |
| Mammalian Cell Lysis Buffer 5X | abcam | ab179835 |
| **Critical Commercial Assays** |  |  |
| CCK-8 kit | MCE | 100266 |
| Annexin V FITC Apop Dtec Kit I | BD Pharmingen | 0076884 |
| Annexin V-APC/7-AAD apoptosis kit | MultiSciences | AP105-100-kit |
| Glucose 6 Phosphate Dehydrogenase Assay Kit (Colorimetric) | abcam | ab102529 |
| NADP/NADPH-Glo Assay | promega | G9081 |
| GSH/GSSG Ratio Detection Assay Kit II (Fluorometric - Green) | abcam | ab205811 |
| NADPH/NADP^+^ quantitation kit | Beyotime | S0179 |
| GSH and GSSG Assay Kit | Beyotime | S0053 |
| Reactive Oxygen Species Assay Kit | Beyotime | S033S |
| HyperScript Ⅲ 1st Strand cDNA Synthesis Kit with gDNA Remover | NovaBio | F3627 |
| 2× S6 Universal SYBR qPCR Mix | NovaBio | Q204 |
| Lipofectamine 3000 Transfection Kit | Invitrogen | 2241260 |
| **Reagent or sources** |  |  |
| Fetal bovine Serum(FBS) | Gibico | 2148169CP |
| Dulbecco’s modified Eagle’s medium(DMEM, | Gibico | 2462637 |
| Mammalian Cell Lysis Buffer | abcam | GR3359014-4 |
| RIPA | NCM Biotech | 20210805 |
| phosphatase and protease inhibitor mix | NCM Biotech | 20201210 |
| Protein A/G Magnetic Beads | MCE | HY-K0202 |
| TRIzol Reagent | Invitrogen | 182805 |
| Crystal violet staining solution | Sangon Biotech | HB16FA0004 |
| Polybrene | Sigma | H9268 |
| **Plasmids and oligos** | | |
| G6PD-OE cDNA | OBIO TECHNOLOGY | HYC3117 |
| G6PD-shRNA | Public Protein/Plasmid Library | PPL01665 |

Table S2 Primer of sequence for RT-qPCR of mRNAs

| Gene | Forward primer | Reverse primer | Gene ID |
| --- | --- | --- | --- |
| β-actin | CACCATTGGCAATGAGCGGTTC | AGGTCTTTGCGGATGTCCACGT | 60 |
| G6PD | CGGCTGTCCAACCACATCTCC | CTGCACCATCTCCTTGCCCAG | 2539 |
| 6PGD | GCATTCCCATGCCCTGTTTTAC | TGGTGTGGATAAACTGCCCTGG | 5226 |
| TAL | TGCCTGTGCTCTCAGCCAAGG | TTCTCCACAGCCATCTGGTCCT | 6888 |
| TKT | CCAAGTGATGGCGTTGCTACAG | TTGTCCGACCTGGAAGTCCTCA | 7086 |
| NADK | GTCCTTTGATGGACGGAAGAGAC | GAGGCTCTCAAACCAGTCGCTC | 65220 |

Table S3 Plasmids and oligos used in the study.

| **Plasmids or oligos** | **Target gene sequence** |
| --- | --- |
| **G6PD-shRNA** | |
| sh-1 | GCCTTCCATCAGTCGGATA |
| sh-2 | GTCGTCCTCTATGTGGAGAAT |
| Sh-3 | CAACAGATACAAGAACGTGAA |
| **G6PD-OE cDNA** | |
| Forward | CGCAAATGGGCGGTAGGCGTG |
| Reverse | AAGAACGGAGCCGGTTGGCG |
