# Supplementary material for: A feedback loop of PPP and PI3K/AKT signal pathway drives regorafenib-resistance in HCC
Source: Cancer Metab. 2023 Dec 18;11:27. doi: 10.1186/s40170-023-00311-5 (PMC10726576; doi:10.1186/s40170-023-00311-5)
Supplement: Supplementary file 2 — Additional file 2: Table S4. Identification of primary metabolites. [file 40170_2023_311_MOESM2_ESM.zip › 1-Additional file 2.docx]

Supplementary Information

A feedback loop of PPP and PI3K/AKT signal pathway drivers regorafenib-resistance in HCC

Huihua Yang^1,2†^, Dahong Chen^1†^, Yafei Wu^1^, Heming Zhou^1^, Wenjing Diao^1^, Gaolin Liu^1*^, Qin Li^1*^

Table S4. Identification of primary metabolites.

| No. | Compounds | Formula | Precursor(g/mol) | Mass  (g/mol) | RT(min) | pathway | Type |
| --- | --- | --- | --- | --- | --- | --- | --- |
| 1 | D-Mannose 6-phosphate | C6H13O9P | 259.0227 | 260.0297 | 0.8922 | Amino sugar and nucleotide sugar metabolism | down |
| 2 | L-Lactic acid | C3H6O3 | 89.0247 | 90.0317 | 1.2188 | Glycolysis / Gluconeogenesis | up |
| 3 | Dihydroxyacetone | C3H6O3 | 89.0246 | 90.0317 | 1.0184 | Glycerolipid metabolism | up |
| 4 | UDP-D-Glucuronic acid | C15H22N2O18P2 | 579.0402 | 580.0343 | 8.5298 | Pentose and glucuronate interconversions | up |
| 5 | UDP-D-glucose | C15H24N2O17P2 | 566.0508 | 566.055 | 1.2271 | Pentose and glucuronate interconversions | down |
| 6 | D-Ribofuranose 5-phosphate | C5H11O8P | 229.0119 | 230.0192 | 1.2243 | Pentose phosphate pathway | down |
| 7 | D-Xylulose 5-phosphate | C5H11O8P | 229.0122 | 230.0192 | 0.9047 | Pentose phosphate pathway | down |
| 8 | 3-Phospho-D-glycerate | C3H7O7P | 150.9818 | 185.9929 | 1.2428 | Pentose phosphate pathway | down |
| 9 | Xanthine | C5H4N4O2 | 151.0262 | 152.0334 | 1.2265 | Purine metabolism | down |
| 10 | Hypoxanthine-9-β-D-Arabinofuranoside | C10H12N4O5 | 557.1357 | 268.081 | 1.3748 | Purine metabolism | down |
| 11 | ADP-ribose | C15H23N5O14P2 | 559.067 | 559.0717 | 1.227 | Purine metabolism | down |
| 12 | Faicar | C10H15N4O9P | 367.0546 | 366.0577 | 1.3706 | Purine metabolism | down |
| 13 | Inosine | C10H12N4O5 | 267.0739 | 268.0808 | 1.3698 | Purine metabolism | down |
| 14 | Xanthosine 5'-(trihydrogen diphosphate) | C10H14N4O12P2 | 445.0165 | 444.0083 | 1.3754 | Purine metabolism | down |
| 15 | 3-Deaza-2'-deoxyadenosine | C11H14N4O3 | 285.0726 | 250.1066 | 1.2607 | Purine metabolism | up |
| 16 | Adenosine monophosphate | C10H14N5O7P | 370.0528 | 347.0631 | 1.2507 | Purine metabolism | up |
| 17 | Adenine | C5H5N5 | 158.0443 | 135.0545 | 1.2258 | Purine metabolism | up |
| 18 | 2'-Deoxyadenosine | C10H13N5O3 | 320.0968 | 251.1018 | 1.8905 | Purine metabolism | up |
| 19 | Bucladesine | C18H24N5O8P | 470.1418 | 469.1362 | 2.3558 | Purine metabolism | up |
| 20 | Thymidine | C10H14N2O5 | 241.0831 | 242.0903 | 1.9786 | Pyrimidine metabolism | down |
| 21 | Uridine | C9H12N2O6 | 243.0625 | 244.0695 | 1.2352 | Pyrimidine metabolism | down |
| 22 | Nicotinic acid | C6H5NO2 | 124.0393 | 123.032 | 1.2206 | Nicotinate and nicotinamide metabolism | down |
| 23 | 2,5-Dihydroxypyridine | C5H5NO2 | 129.0655 | 111.032 | 0.8474 | Nicotinate and nicotinamide metabolism | up |
| 24 | Dihydroxyethyl-TPP | C14H23N4O9P2S | 995.1226 | 485.0661 | 8.5373 | Thiamine Metabolism | up |
| 25 | Thiamine monophosphate | C12H17N4O4PS | 365.0499 | 344.0708 | 1.3678 | Thiamine Metabolism | down |
