# Supplementary material for: A feedback loop of PPP and PI3K/AKT signal pathway drives regorafenib-resistance in HCC
Source: Cancer Metab. 2023 Dec 18;11:27. doi: 10.1186/s40170-023-00311-5 (PMC10726576; doi:10.1186/s40170-023-00311-5)
Supplement: Supplementary file 3 — Additional file 3: Figure S1. Multivariate statistical analysis and metabolic profiling. (A) total ion chromatogram(TIC) of QCsamples. (B) PCA analysis of QC. Figure S2. Multivariate modelling of LC-MS data. (A) PCA score plot in positivemode. (B) OPLS-DA score plot in positive mode. (C) Validation of positive mode.(D) Permutation tests of positive mode. (E) PCA score plot in negative mode. (F)OPLS-DA score plot in negative mode. (G) Validation of negative mode. (H) Permutationtests of negative mode. Figure S3. Differentialmetabolites variation. (A) The volcano plots(red spots represents increasedmetabolites, red spots represents decreased metabolites and gray spotsrepresents not significant metabolites); (B) cluster analysis of heat map(colorred represents increased metabolites, and the color green represents decreased metabolites).The selection of differential metabolites was according to variable importancein projection(VIP) based on OPLS-DA, pvalue fromStudent’s t-test and fold change. Peaks with VIP≥1, P<0.05，foldchange≥ 2 and fold change≤ 0.5 were included in as differential metabolites. Figure S4. Heat map of cluster analysisof potential biomarkers. Color red represents increased metabolites, and thecolor green represents decreased metabolites. Figure S5. G6PD inhibition the effect of regorafenib onregorafenib-resistant cells in HCC. (A) Cellviability were detected on Huh7 and Huh7-RR cells which were treated with or withoutregorafenib(6 μM), 6AN(30 μM) or the combination of regorafenib(6 μM) and 6AN(30 μM) for 48 h, n=3. (B) Cellviability were detected on G6PD overexpressed cells and it’s counterparts andcells which were treated with or without regorafenib(6 μM), 6AN(30 μM) or thecombination of regorafenib(6 μM) and 6AN(30 μM) for 48 h, n=3. Figure S6. PI3K/AKTsignaling pathway and NADK were involved in the mechanism of G6PD inducedregorafenib-resistance in HCC. (A) Protein levels of PI3K(PI3Kinase p85), p-PI3K(Phospho-PI3-kinase p85-α/γ(Tyr467/199)), AKT(pa [file 40170_2023_311_MOESM3_ESM.doc]

Supplementary Information

A feedback loop of PPP and PI3K/AKT signal pathway drivers regorafenib-resistance in HCC

Huihua Yang^1,2†^, Dahong Chen^1†^, Yafei Wu^1^, Heming Zhou^1^, Wenjing Diao^1^, Gaolin Liu^1*^, Qin Li^1*^


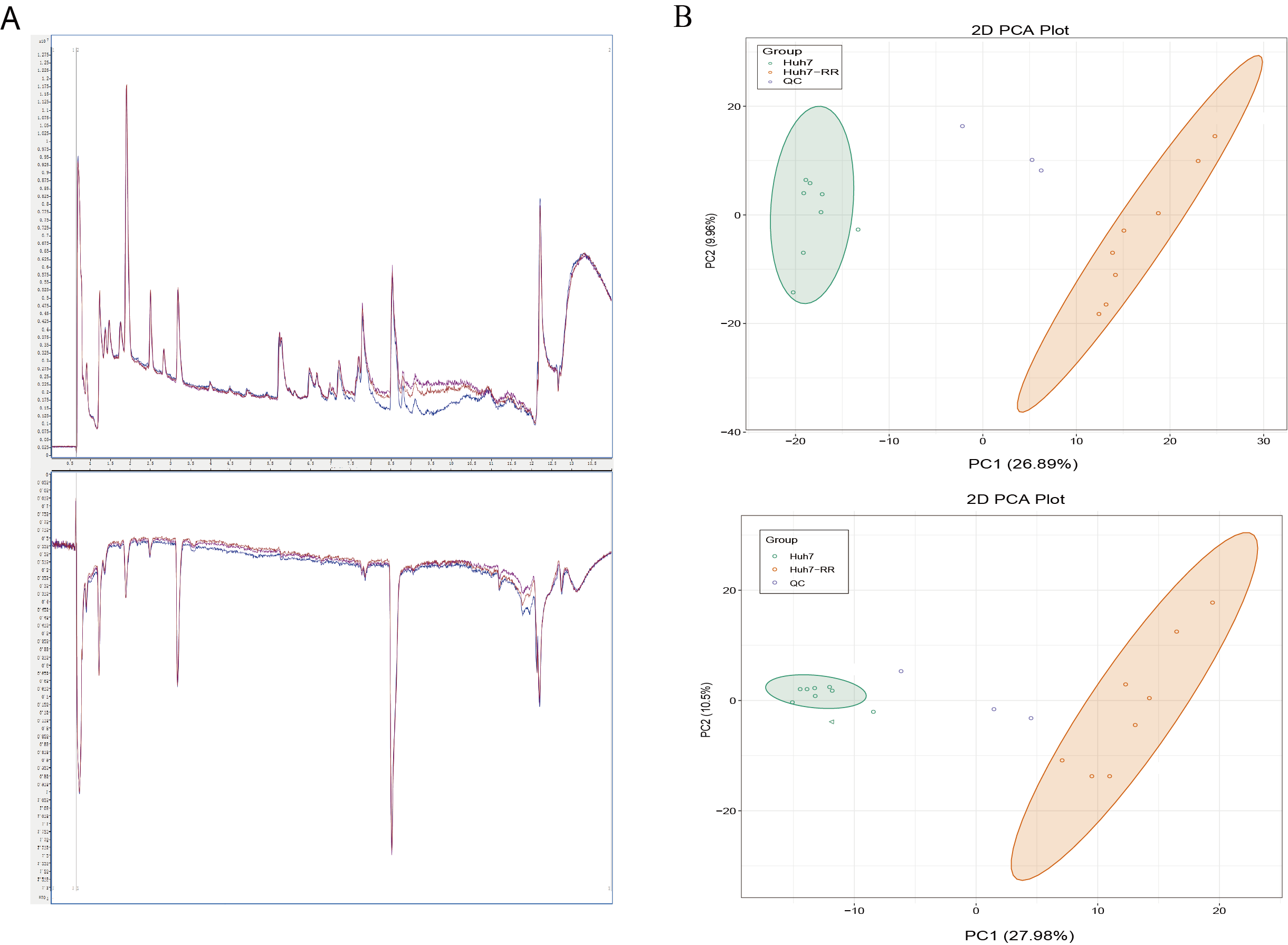


Figure S1. Multivariate statistical analysis and metabolic profiling. (A) total ion chromatogram(TIC) of QC samples. (B) PCA analysis of QC.


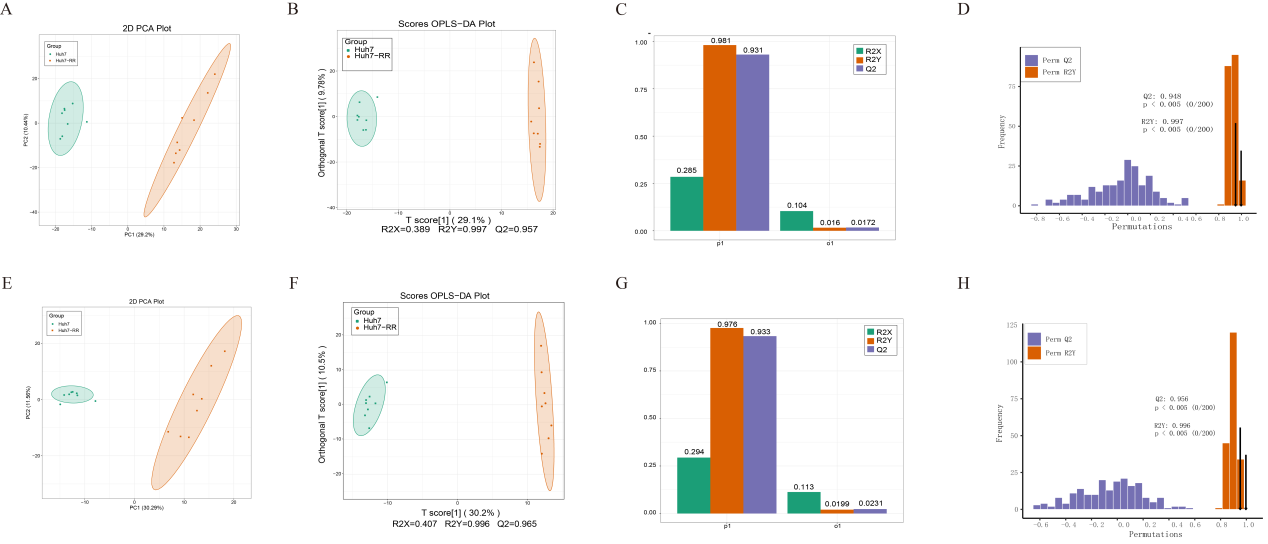


Figure S2. Multivariate modelling of LC-MS data. (A) PCA score plot in positive mode; (B) OPLS-DA score plot in positive mode.(C) Validation of positive mode. (D) Permutation tests of positive mode. (E) PCA score plot in negative mode. (F) OPLS-DA score plot in negative mode.(G) Validation of negative mode. (H) Permutation tests of negative mode.


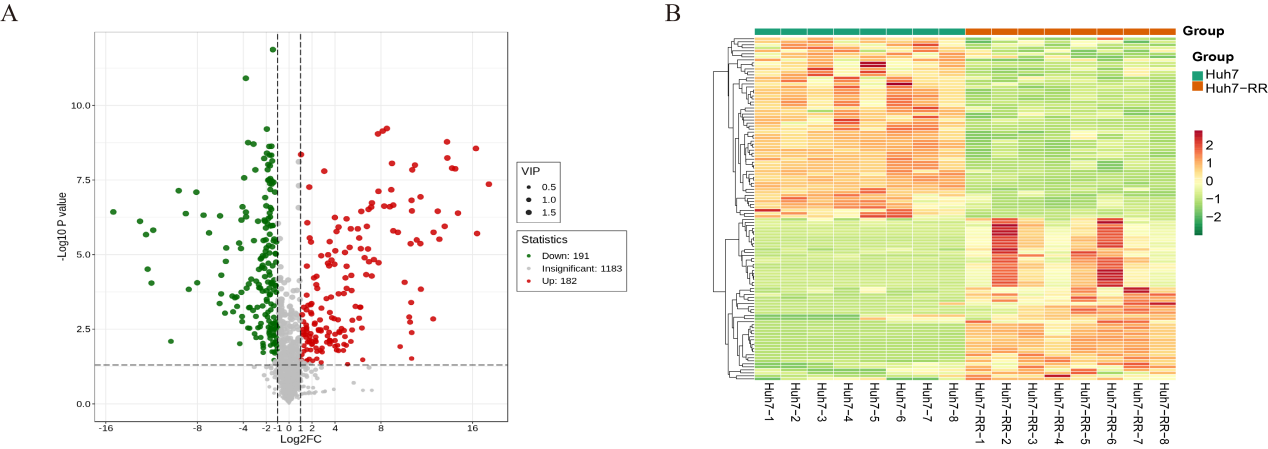


Fig. S3. Differential metabolites variation. (A) The volcano plots(red spots represents increased metabolites, red spots represents decreased metabolites and gray spots represents not significant metabolites). (B) cluster analysis of heat map(color red represents increased metabolites, and the color green represents decreased metabolites). The selection of differential metabolites was according to variable importance in projection(VIP) based on OPLS-DA, p value from Student’s t-test and fold change. Peaks with VIP≥1, P<0.05，fold change≥ 2 and fold change≤ 0.5 were included in as differential metabolites.


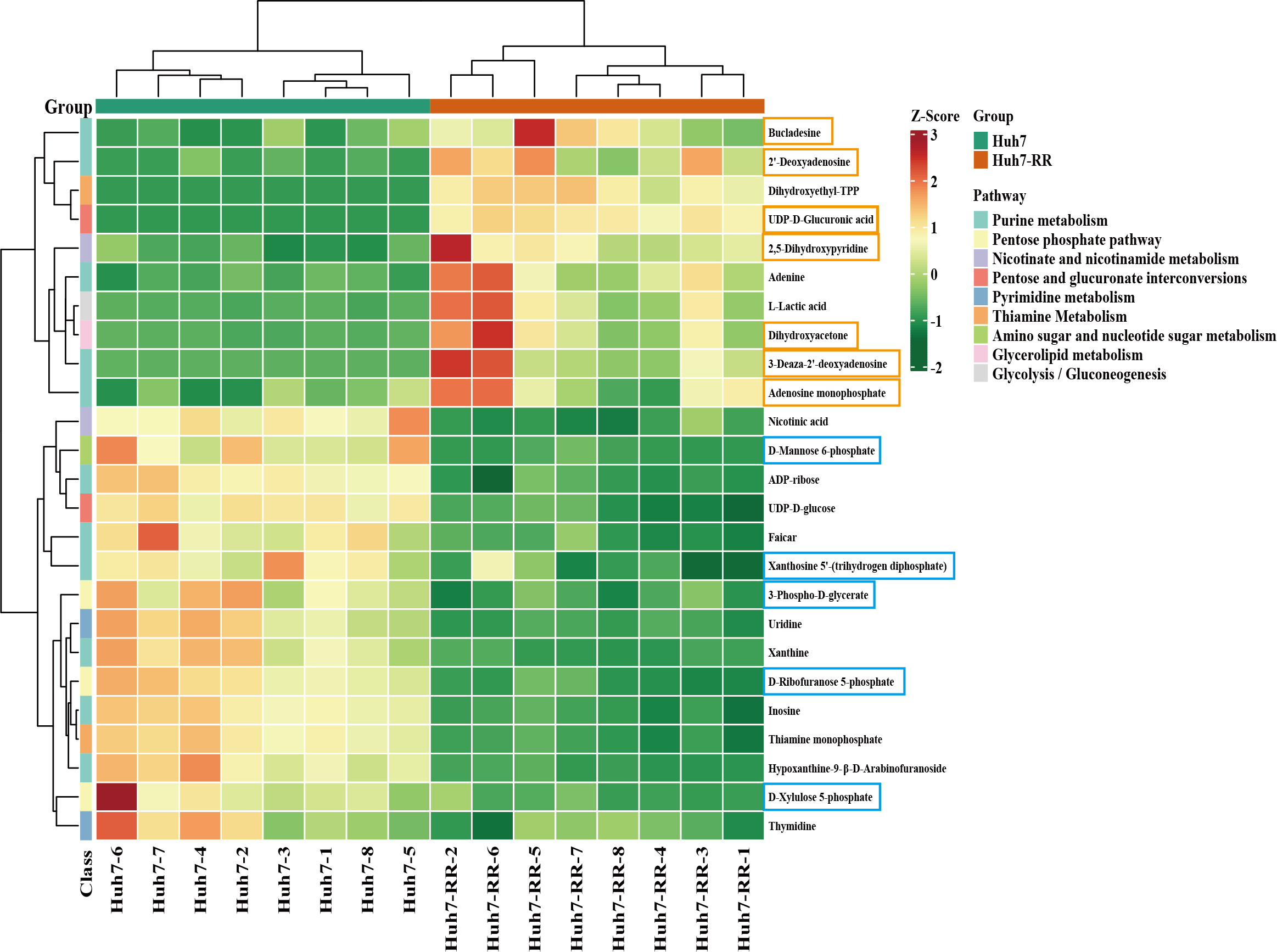


Fig. S4. Heat map of cluster analysis of potential biomarkers.Color red represents increased metabolites, and the color green represents decreased metabolites.


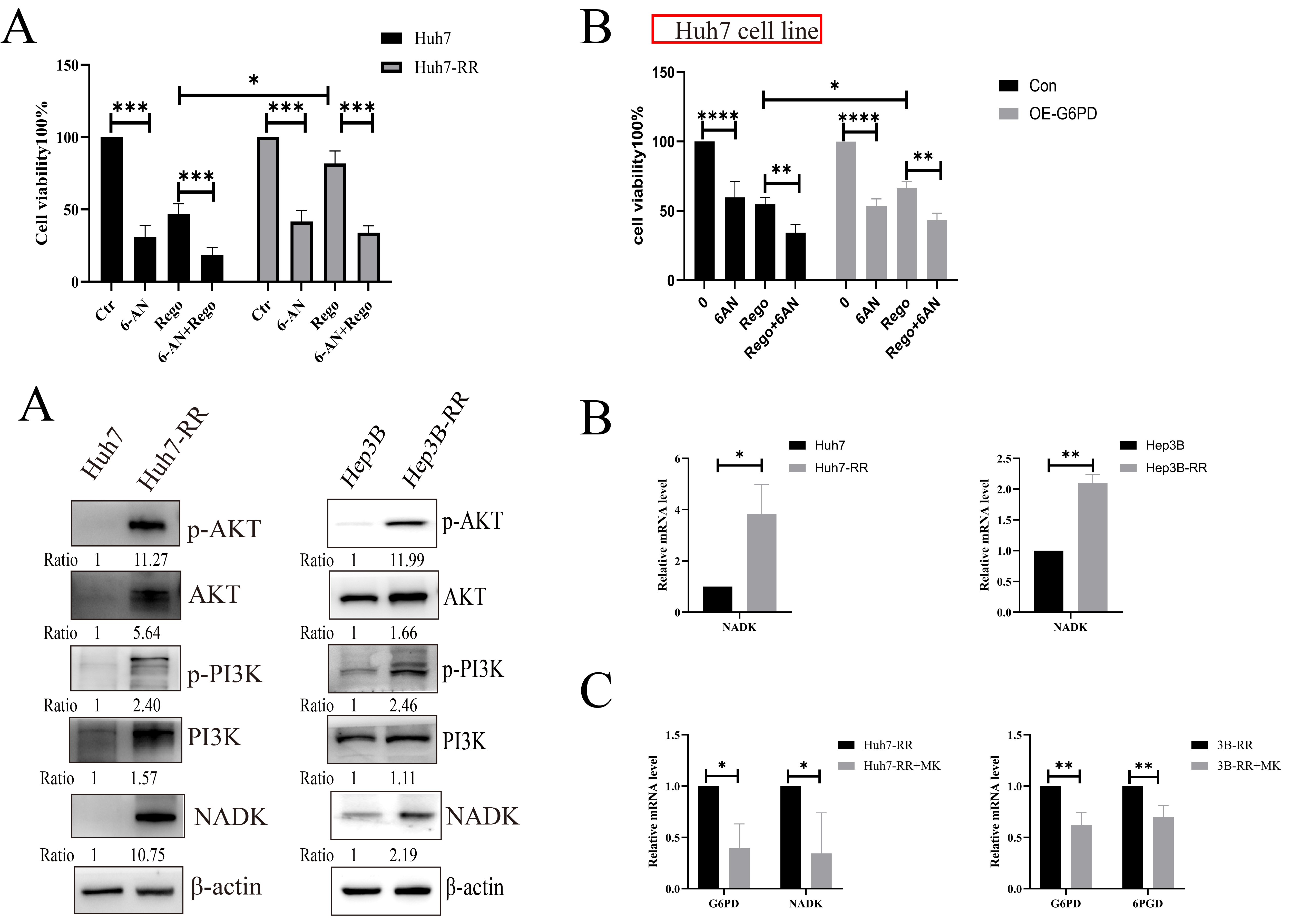


Fig. S5. G6PD inhibition the effect of regorafenib on regorafenib-resistant cells in HCC. (A) Cell viability were detected on Huh7 and Huh7-RR cells which were treated with or without regorafenib(6 μM), 6AN(30 μM) or the combination of regorafenib(6 μM) and 6AN(30 μM) for 48 h, n=3. (B) Cell viability were detected on G6PD overexpressed cells and it’s counterparts and cells were treated with or without regorafenib(6 μM), 6AN(30 μM) or the combination of regorafenib(6 μM) and 6AN(30 μM) for 48 h, n=3.^*^*P*<0.05, ^**^*P*<0.01, ^***^*P*<0.001, mean ±SD.


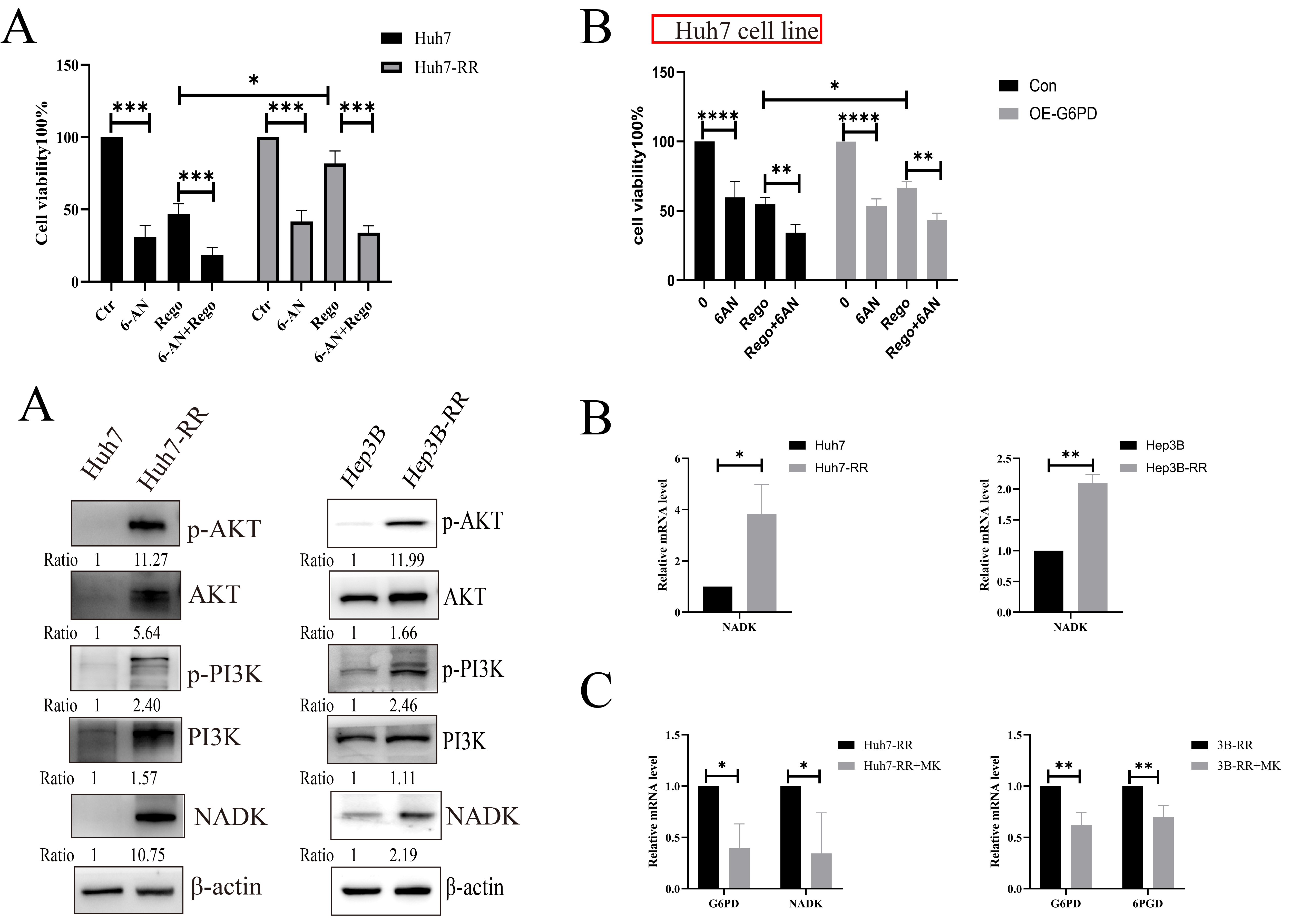


Fig. S6. PI3K/AKT signaling pathway and NADK were involved in the mechanism of G6PD induced regorafenib-resistance in HCC. (A) Protein levels of PI3K(PI3 Kinase p85), p-PI3K(Phospho-PI3-kinase p85-α/γ(Tyr467/199)), AKT(pan), p-AKT(ser473) and NADK were detected by western blot in Huh7 and Huh7-RR (left, n=3), Hep3B and Hep3B-RR(right, n=3). (B) The mRNA levels of NADK, n=3. (C) The effects of PI3K/AKT signal pathway inhibition by MK-2206(MK, 10 μM) on the mRNA levels of G6PD and NADK in regorafenib-resistant cells, n=3.^*^*P*<0.05, ^**^*P*<0.01, ^***^*P*<0.001, mean ±SD.
